# Relationship of Digital Game Addiction with Aggression and Anger in the Post COVID-19 Era: A Systematic Review and Meta-Analysis

**DOI:** 10.17533/udea.iee.v43n2e13

**Published:** 2025-07-25

**Authors:** Kaushal Nagar, Anil Kumar Patidar, Shiv Kumar Mudgal, Rakhi Gaur, Vipin Patidar

**Affiliations:** 1 . M.Sc. Tutor/clinical Instructor. Email: kaushalnagar891@gmail.com. https://orcid.org/0009-0001-3222-6296 All India Institute Of Medical Sciences India kaushalnagar891@gmail.com; 2 . M.Sc. Assistant Professor. Email: anilpatidar.nur@charusat.ac.in. https://orcid.org/0000-0003-1474-6041 Charotar University Of Science & Technology India anilpatidar.nur@charusat.ac.in; 3 . Ph.D, Associate Professor. Email: shiv.nur@aiimsdeoghar.edu.in. https://orcid.org/0000-0002-8062-0589 All India Institute Of Medical Sciences India shiv.nur@aiimsdeoghar.edu.in; 4 . Ph.D, Assistant Professor. Email: rakhi.nur@aiimsdeoghar.edu.in. https://orcid.org/0000-0003-0835-4383 All India Institute Of Medical Sciences India rakhi.nur@aiimsdeoghar.edu.in; 5 . M.Sc. Tutor/clinical Instructor. Email: Vipin.nur@aiimsdeoghar.edu.in. Corresponding Author. https://orcid.org/0000-0003-4595-9859 All India Institute Of Medical Sciences India Vipin.nur@aiimsdeoghar.edu.in; 6 . College of Nursing, All India Institute of Medical Sciences, Raebareli, Uttar Pradesh, India. All India Institute Of Medical Sciences College of Nursing All India Institute of Medical Sciences Raebareli Uttar Pradesh India; 7 . Department of Nursing, CHARUSAT Campus, Charotar University of Science and Technology, Changa, Gujarat, India. Charotar University Of Science & Technology Department of Nursing CHARUSAT Campus Charotar University of Science and Technology Changa Gujarat India; 8 . College of Nursing, All India Institute of Medical Sciences, Deoghar, Jharkhand, India. All India Institute Of Medical Sciences College of Nursing All India Institute of Medical Sciences Deoghar Jharkhand India

**Keywords:** addiction disorder, aggression, anger, meta-analysis, COVID-19., trastorno de adicción a internet, agresión, ira, metaanálisis, COVID-19., transtorno de adição à internet, agressão, ira, metanálise COVID-19.

## Abstract

**Objectives.:**

To evaluate the relationship of digital gaming addiction with aggression and anger behavior among people.

**Methods.:**

This meta-analysis and systematic review was conducted using PRISMA and MOOSE guidelines to find articles in the databases PubMed, Scopus, Embase, and EBSCO. The evaluation comprised ten studies with 11,259 individuals. Researchers systematically extracted data on aggression, anger, and gaming addiction. The meta-analysis evaluated heterogeneity and pooled correlations using random-effects models. The study protocol was registered with PROSPERO under the registration number CRD42025642494.

**Results.:**

Addiction to digital games was found to be strongly correlated with aggression (r = 0.531, 95% CI [0.226, 0.836]) and moderately with anger (r = 0.348, 95% CI [0.177, 0.518]). Regional analysis revealed that Saudi Arabia study had the strongest correlation (β = 1.004, *p*<0.001), whereas Italy, Nepal, Singapore, and Turkey studies had comparatively lower correlations. Anger consequences were also found to be moderated correlations by age, with younger adolescents experiencing more negative consequences (β = -0.0696, *p*=0.049).

**Conclusion.:**

The meta-analysis demonstrates significant positive correlations between digital game addiction, aggression, and anger, highlighting the importance of nurse-led interventions in vulnerable groups in order to promote the reduction of the negative consequences of digital addiction.

## Resumen

**Objetivo.:**

Evaluar la relación de la adicción a los juegos digitales con la agresión y el comportamiento de ira.

**Métodos.:**

Esta revisión sistemática con meta-análisis se realizó utilizando las directrices de las guías PRISMA y MOOSE para encontrar artículos en las bases de datos PubMed, Scopus, Embase y EBSCO. La evaluación comprendió diez estudios con 11 259 individuos. Los investigadores extrajeron sistemáticamente datos sobre agresividad, ira y adicción al juego. El meta-análisis evaluó la heterogeneidad y agrupó las correlaciones mediante modelos de efectos aleatorios. El protocolo del estudio se registró en PROSPERO con el número de registro CRD42025642494.

**Resultados.:**

Se observó que la adicción a los juegos digitales estaba fuertemente correlacionada con la agresividad (r = 0.531; IC del 95%: [0.226; 0.836]) y moderadamente con la ira (r = 0.348; IC del 95%: [0.177; 0.518]). El análisis regional reveló que un estudio de Arabia Saudí tenía la correlación más fuerte (β = 1.004, *p*<0.001), mientras que otros estudios realizados en Italia, Nepal, Singapur y Turquía tenían correlaciones comparativamente más bajas. Las consecuencias de la ira también se correlacionaron moderadamente con la edad, teniendo los adolescentes más jóvenes las consecuencias más negativas (β = -0.0696, *p*=0.049).

**Conclusión.:**

El meta-análisis muestra correlaciones positivas y significativas entre la adicción a los juegos digitales, la agresión y la ira, lo que pone de relieve la importancia de realizar intervenciones lideradas por enfermería en los adolescentes vulnerables con fin de promover la reducción de las consecuencias negativas de la adicción digital.

## Resumo

**Objetivo.:**

Avaliar a relação entre a adição a jogos digitais e o comportamento de agressão e raiva.

**Métodos.:**

Esta meta-análise com revisão sistemática foi conduzida usando as diretrizes PRISMA e MOOSE para buscar artigos nas bases de dados PubMed, Scopus, Embase e EBSCO. A avaliação compreendeu dez estudos com 11.259 indivíduos. Os pesquisadores extraíram sistematicamente dados sobre agressão, raiva e vício em jogos. A meta-análise avaliou a heterogeneidade e as correlações agrupadas usando modelos de efeitos aleatórios. O protocolo do estudo foi registrado no PROSPERO sob o número de registro CRD42025642494.

**Resultados.:**

O vício em jogos digitais foi fortemente correlacionado com a agressão (*r*=0.531; IC de 95%: [0.226; 0.836]) e moderadamente correlacionado com a raiva (*r*=0.348; IC de 95%: [0.177; 0.518]). A análise regional revelou que um estudo da Arábia Saudita apresentou a correlação mais forte (β =1.004, *p*<0.001), enquanto outros estudos conduzidos na Itália, Nepal, Cingapura e Turquia apresentaram correlações comparativamente menores. As consequências da raiva também foram moderadamente correlacionadas com a idade, com adolescentes mais jovens apresentando as consequências mais negativas (β = -0.0696, *p*=0.049).

**Conclusão.:**

A meta-análise mostra correlações positivas e significativas entre vício em jogos digitais, agressão e raiva, destacando a importância de intervenções focadas em adolescentes vulneráveis a fim de promover a redução das consequências negativas da dependência digital.

## Introduction

Gaming is a widely popular and rapidly growing leisure activity worldwide, with an estimated 2.7 billion gamers in 2020.^1^ While gaming offers entertainment and social interaction, excessive gaming has been linked to behavioural concerns, including anger and aggression.^1^ According to ICD-11, disordered gaming is characterized by a pattern of gaming behaviour (digital or video) where individuals lose control, prioritize gaming over other activities, and persist despite negative consequences. Similarly, digital game addiction refers to excessive and impulsive gaming that leads to social or emotional problems, accompanied by difficulty in regulating this behaviour.^2^^,^^3^ The COVID-19 pandemic increased digital gaming due to lockdowns, remote learning, and social restrictions. While gaming provided entertainment and social connection, concerns grew over excessive gaming and its psychological and behavioural effects on individuals.^4^^-^^7^ Even after the pandemic, many people continue the gaming habits they developed during lockdowns and this has raised worries about the long-term effects of digital game addiction.

Playing digital games is generally considered normal and can even have positive effects, such as providing emotional relief, relaxation, improved leisure time utilization, and enhanced problem-solving skills.^8^ However, excessive and uncontrolled gaming has led to the emergence of the term 'game addiction,' raising serious concerns worldwide due to its associated negative consequences.^9^ The significant prevalence of gaming disorder highlights a growing public health concern, particularly due to its potential association with increased anger and aggression.^10^^,^^11^ Aggression and anger are essential areas of exploration in gaming research. Anger is a heightened emotional response triggered by frustration, in-game losses, perceived failure, or obstacles encountered during gameplay.^9^ In the context of digital game addiction, excessive gaming can intensify these emotional reactions, leading to difficulties in emotional regulation. When anger remains uncontrolled, it may escalate into aggression, which is characterized as a behaviour directed toward another individual with the immediate intent to cause harm.^10^ Understanding the relationship between digital game addiction, anger, and aggression is essential for addressing its psychological and behavioral consequences.^12^^,^^13^ Previous researches have consistently demonstrated a relationship between gaming and the manifestation of aggression and anger behaviors.^14^^-^^19^ The underlying mechanism of this relationship assumes that aggression and anger contribute to excessive gaming. One perspective posits that players tend to select games that align with their pre-existing characteristics, including anger and aggression.^20^^-^^21^

As the world moves towards normalcy following the COVID-19 pandemic, it is more important than ever for healthcare professionals, especially nurses to understand the long-term behavioral and emotional effects of digital gaming disorders. Nurses frequently serve as the first point of contact for people exhibiting emotional dysregulation and aggression, as well as for their management and counseling. The possible association between excessive digital gaming with aggression and anger is a serious public health issue that has broad ramifications for nursing practice in a variety of settings, such as educational institutions, community health programs, mental health services, and acute care hospitals. This study examines the relationship of digital game addiction with aggression and anger in post COVID era, providing insights that may help nurses with patient evaluation, early intervention techniques, psychosocial support, and health education.

## Methods

Type of study. This systematic review and meta-analysis combined and analysed existing studies to investigate how digital game addiction correlates with aggression and anger among individuals after the COVID-19 pandemic. It followed strict guidelines (MOOSE and PRISMA) and registered with PROSPERO (CRD42025642494)."

Information Resources and the Search Equation. A systematic search was conducted on studies published during and after COVID (published after year 2020) using four databases: PubMed, Scopus, Embase, and EBSCO. The search strategy incorporated Mesh terms and general keywords related to online gaming addiction, including “Digital Game Addiction” OR “Mobile game addiction” OR “Gaming overuse” OR “Video game dependency” OR “Internet gaming dependency” OR “Internet gaming addiction” OR “Online gaming dependency”. Additionally, terms associated with anger, such as “anger” OR “rage” OR “Road rage” OR “fury” OR “wrath” as well as aggression-related terms like “aggressiveness” OR “Aggressive behaviour” OR “aggression” OR “violent behaviour” OR “irritating” were included. To find all relevant studies, the researchers also checked the reference lists of the included papers. All identified studies were then managed in Rayyan software, where duplicates were removed. Subsequently, two researchers independently reviewed titles and abstracts to determine which studies met the predefined criteria for inclusion. The shortlisted papers were then reviewed in full text, which was also performed separately by two authors. Where there was disagreement, a third author was consulted to make a final decision.

Study selection. Studies were included if they met the following criteria: a) original, peer-reviewed research article published in English, b) observational or cross-sectional studies focusing on individuals published during and after COVID-19, c) studies examining the relationship of digital game addiction with aggression and anger among individuals. Studies were excluded if they had fewer than 50 participants, duplicate observational studies, or lacked sufficient individual-level data related to aggression and anger. Only specific study designs were included, with experimental studies, case-control studies, case reports, editorials, commentaries, clinical practice guidelines, opinions, and reviews being excluded.

Codification of the findings. Data including authors, publication year, country, anger and aggression measurement instruments, and risk of bias, were independently extracted by two reviewers into a pre-defined Microsoft Excel file. Participants data, including age (mean, SD, or range) and gender distribution (male-to-female ratio), were also tabulated. Primary findings, including sample size, gaming addiction scores, aggression scores, and anger scores (and their means and standard deviations), and correlations (r) between gaming addiction with aggression and anger were examined. Any discordance in study selection was resolved by a third reviewer. If there were missing data necessary for inclusion, the authors of the studies were emailed for clarification.

Risk of Bias. To evaluate the risk of bias in studies examining the relationship of digital game addiction with aggression and anger in individuals, two researchers independently used the Joanna Briggs Institute tool to assess the methodological quality of prevalence studies.^22^ A third researcher resolved any disagreements. The tool scored studies as low, moderate, or high quality based on nine criteria."

Statistical Analysis. To better understand the effects of digital game addiction with aggression and anger among individuals after COVID-19, we conducted a meta-analysis by examining its relationship with aggression and anger behaviours. Given the variability among studies, we used a random-effects model to ensure a more accurate overall estimate, with results presented using 95% confidence intervals (CIs). To assess differences between studies, we measured heterogeneity using the Cochran’s Q statistic and I² test, which helped us determine how much variation existed across the included research. Additionally, we performed Meta-ANOVA (moderation analysis) based on country and age to explore potential regional differences in the effects of digital game addiction with aggression and anger behaviours. All statistical analyses were conducted using R software (version 4.2.3). Specifically, we used the "predict" function to estimate the overall pooled correlation of digital game addiction with aggression and anger and the "rma" function to calculate the moderation analysis based on country and age across studies.

## Results

### Search Results

**Figure 1:**
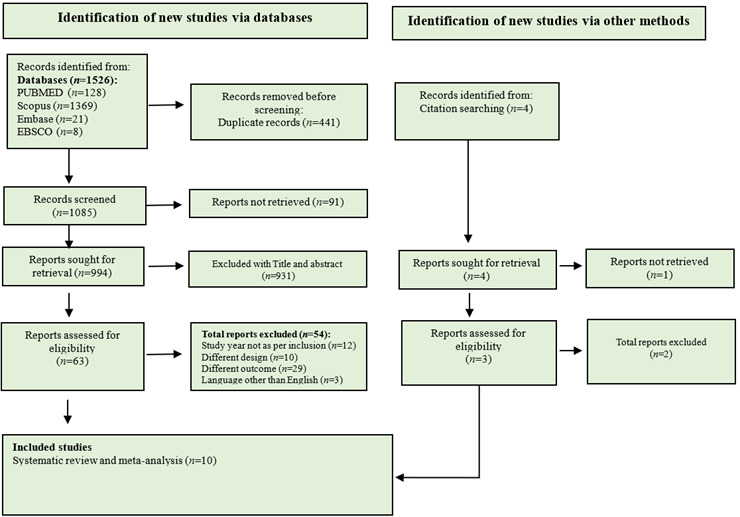
PRISMA flow chart

We systematically identified studies meeting the inclusion criteria using PRISMA guidelines (Figure 1). Initial database searches yielded 1526 records, with 441 duplicates removed in Rayyan software. After screening titles/abstracts of 994 studies, 931 were excluded for irrelevance. Full-text reviews of the remaining 63 studies led to the exclusion of 53 studies. One additional study was identified through citation searching, resulting in a final inclusion of 10 studies (total participants: 11,259) for systematic review and meta-regression.

### Study Characteristics

The analysis included 10 studies with a total of 11,259 participants from eight countries. Turkey contributed the largest number, with five studies,^23^^,^^24^^,^^30^^-^^32^ followed by China,^27^ Saudi Arabia,^28^ Italy,^25^ Singapore,^26^ and Nepal^29^ each with one (Table 1). The ages of the participants ranged widely, from 10.57 to 29.6 years, spanning adolescents and young adults. Sample sizes also varied widely, from 52 to 7,318 participants. Addiction to digital games was predominantly assessed using various assessment measures among the 15 studies, viz., Internet Gaming Disorder Scale-Short Form (IGDS9-SF; 4 studies), Digital Game Addiction Scale for Children (CDGAS; 2 studies), Gaming Addiction Scale (GAS; 2 studies), Digital Game Addiction Scale (DGAS-7; 1 study), and Internet Gaming Disorder Test (IGD-20; 1 study). Incidence of gaming addiction across the studies varied between 36.43±15.89 to 57.45±22.99.

Aggression was assessed in nine studies,^23^^-^^31^ primarily using the Buss-Perry Aggression Questionnaire (BPAQ; 7 studies), while the Sahin Aggression Scale (SA; 1 study) and the Impulsive/Premeditated Aggression Scale (IPAS; 1 study) were also used. The reported mean aggression scores varied widely, ranging from 19.60±5.36 to 119.69±13.58, indicating differences in aggression intensity across populations. Anger was measured in three studies^23^^,^^29^^,^^32^ using the Trait Anger and Anger Expression Style Scale and the Anger subscale, with mean scores between 19.05±5.39 and 22.81±6.81. Compared to aggression, fewer studies reported data on anger outcomes. The correlation between gaming addiction and aggression varied across studies, ranging from r = 0.24 to r = 0.92, with Turkish studies accounting for 40% of the total sample. Meanwhile, the correlation between gaming addiction and anger ranged from r =0.18 to r = 0.45.

### Effect Sizes and Homogeneity Test

**Figure 2:**
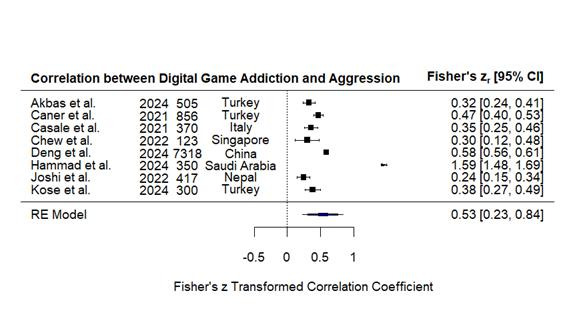
Pooled correlation between digital game addiction and aggression

**Figure 3:**
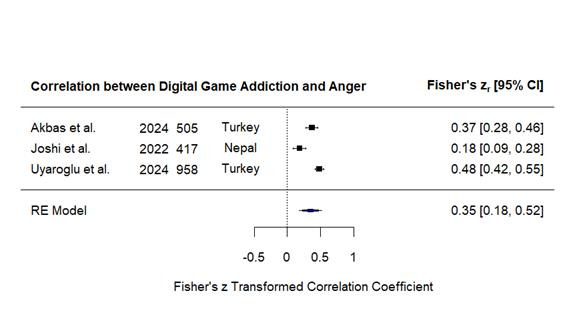
Pooled correlation between digital game addiction and anger.

Eight studies^23^^-^^30^ that tested the correlation between digital game addiction and aggression (Figure 2) and three studies^23^^,^^29^^,^^32^ that tested its correlation with anger (Figure 3) were used in the meta-regression analysis and involved 10,239 and 1,880 participants, respectively. Homogeneity test revealed substantial heterogeneity for aggression (Q = 481.45, *p* < 0.001, I² = 99.25%) and anger (Q = 25.88, *p* < 0.001, I² = 92.32%), implying heterogeneity likely due to differences in measurement scales, cultural settings, and sample sizes. To control for this high heterogeneity, random-effects models were used. The correlation analysis found a high, significant correlation between digital game addiction and aggression (r = 0.531, 95% CI [0.226, 0.836], Z = 3.411, *p* < 0.001) and a moderate, significant correlation between digital game addiction and anger (r = 0.348, 95% CI [0.177, 0.518], Z = 4.006, *p* < 0.001). 

**Table 1:** Summary table of included studies

Author (year)	Country	Age [Mean (±) SD]	Sample size (N)	Male: female (n)	Gaming addiction scale	Anger scale	Aggression scale	Gaming addiction [Mean (±) SD]	Anger [Mean (±) SD]	Aggression [Mean (±) SD]	r (addiction vs. anger)	r (addiction vs. aggression)	Quality
Akbas (2024)	Turkey	16.33±1.48	505	247:258	GAS	Anger subscale	BPAQ	36.43±15.89	19.05±5.39	119.69±13.58	0.352	0.314	High
Caner (2021)	Turkey	15.4±1.32	856	291:565	DGAS-7	NA	BPAQ	11.54±4.79	NA	64.01±18.87	NA	0.436	High
Casale (2021)	Italy	29.63±7.64	370	270:100	IGD9-SF	NA	BPAQ	1.85±0.67	NA	64.05±23.12	NA	0.34	Moderate
Chew (2022)	Singapore	25.02±5.34	123	52:71	IGDS9-SF	NA	BPAQ	17.93±5.92	NA	76.06±20.39	NA	0.29	High
Deng (2024)	China	15.8±1.45	7318	3891:3427	IGD-20	NA	IPAS	38.35±13.15	NA	69.51±22.19	NA	0.526	High
Hammad (2024)	Saudi Arabia	21.30±4.96	350	186:164	IGDS9-SF	NA	BPAQ	27.23±6.087	NA	28.85±6.88	NA	0.92	High
Joshi (2022)	Nepal	17±1.411	417	278:139	IGDS9-SF	Not mentioned	BPAQ	NA	19.88±5.33	83.79±17.98	0.182	0.239	High
Kose (2024)	Turkey	10.57±0.49	300	144:156	CDGAS	NA	SA	57.45±22.99	NA	19.60±5.36	NA	0.363	High
Tunctruck (2023)	Turkey	14.3±1.7	62	62:0F	GAS	NA	BPAQ	52.0±4.0	NA	89.7±25.8	NA	Not mentioned	High
Koçak Uyaroğlu (2024)	Turkey	13.44±0.50	958	456:502	CDGAS	TAAES	NA	56.74±19.02	22.81±6.81	NA	0.448	NA	High

Note: *n=*number of participants; SD=standard deviation; GAS=Gaming Addiction Scale; DGAS-7=Digital Game Addiction Scale-7; IGD9-SF=Internet Gaming Disorder scale short form; IGD-20=Internet Gaming Disorder Test; IGUESS=Internet Game Use-Elicited Symptom Screen; CDGAS =Digital Game Addiction Scale for Children; TAAES=Trait Anger and Anger Expression Style Scale; BPAQ=Buss-Perry Aggression Questionnaire; SA= Sahin Aggression Scale; IPAS=Impulsive/Premeditated Aggression Scale; r= correlation; NA= Not available.

### Moderator analysis

A random-effects meta-regression was conducted to assess the moderating role of moderator (country and age**)**on the relationship between gaming addiction with aggression and anger. Country-level moderation analyses (Table 2) through meta-ANOVA revealed significant effects between digital game addiction and aggression with substantial variation across regions as Saudi Arabia exhibited the strongest positive association (β=1.004, p<0.001), suggesting cultural or methodological factors amplify the gaming addiction-aggression relationship, while Italy (*β*= -0.2305, *p*= 0.029), Nepal (*β*= -0.3409, *p*= 0.001), Singapore (*β*= -0.2860, *p*= 0.027), and Turkey (*β*= -0.1891, *p*= 0.017) showed significant negative effects, indicating weaker relationships compared to the reference highlighting regional consistency in some contexts. Country-level moderation analyses (Turkey vs. Nepal) with anger outcomes was marginally significant (*β*= 0.1841, *p*= 0.033). Turkey demonstrated a stronger positive association (*β*= 0.2450, *p*= 0.018) highlighting regional disparities.

**Figure 4:**
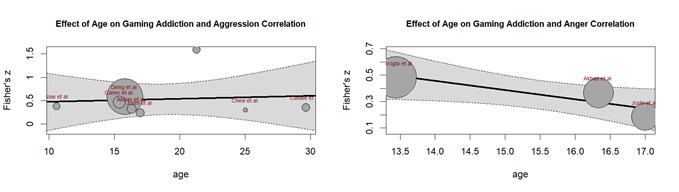
Age wise moderation analyses of digital game addiction with aggression and anger

Age wise moderation analyses (Figure 4) between digital game addiction and aggression revealed a non-significant moderating impact (β = 0.4116, *p* = 0.478) with no linear trends. It showed significant residual heterogeneity (I^2^ = 99.37%, QE = 476.49, *p* < 0.001), indicating that the moderator (age) could not account for almost all of the variability. (β = 0.0063, *p* = 0.830). Age wise moderation analyses with anger (Figure 4) have limited data (k=3) precluded meaningful meta-regression. A visual inspection of the figure revealed a unique pattern of decreasing correlation strength as age advances. Anger and gaming addiction were more strongly correlated among younger adolescents and the correlation decreased by the latter stages of adolescence. The intercept was significant (β = 1.4303,*p* = 0.009), although age showed a significant negative moderating impact (β = -0.0696, *p* = 0.049). The moderators' overall test revealed significance (QM = 3.87, *p* = 0.049), indicating that age explains an element of the variation in the relationship between anger and gaming addiction.

**Table 2:** Country-level moderation analyses through meta-ANOVA of digital game addiction with aggression and anger

Variable	Moderator (Country)	Estimate	95% CI	SE	z-value	***p*-value**
Digital game addiction and Aggression	Intercept (Reference)	0.5846	[0.4567, 0.7125]	0.0653	8.9570	<0.001
	Italy	-0.2305	[-0.4371, -0.0239]	0.1054	-2.1871	0.029
	Nepal	-0.3409	[-0.5446, -0.1372]	0.1039	-3.2804	0.001
	Saudi Arabia	1.0044	[0.7964, 1.2124]	0.1061	9.4636	<0.001
	Singapore	-0.2860	[-0.5394, -0.0326]	0.1293	-2.2123	0.027
	Turkey	-0.1891	[-0.3449, -0.0333]	0.0795	-2.3791	0.017
Digital game addiction and Anger	Intercept (Reference)	0.1841	[0.0149, 0.3532]	0.0863	2.1327	0.033
	Turkey	0.2450	[0.0422, 0.4478]	0.1035	2.3681	0.018

Note: CI=Confidence interval; SE=Standard error;

### Publication Bias Assessment

**Figure 5:**
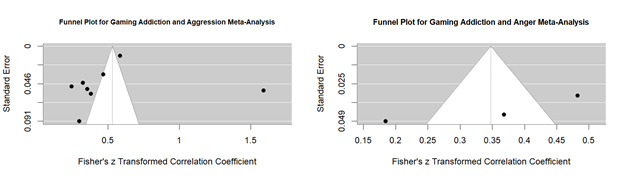
Funnel plot for gaming addiction with aggression and anger meta-analysis

The evaluation of publication bias using Egger's regression test revealed no significant asymmetry in the funnel plot for Gaming Addiction and Aggression Meta-Analysis shown in Figure 5 (-0.242, p = 0.808), indicating a low likelihood of publication bias influencing the meta-analytic results. The limit estimate (effect size as standard error approaches zero) was b = 0.626 (95% CI [-0.211, 1.463]), suggesting that even in the absence of small-study effects, the pooled effect size remains consistent with the observed results. Egger's regression test for funnel plot asymmetry between Gaming addiction and anger (Figure 5) revealed a significant publication bias (z = - 2.466, p = 0.013), indicating asymmetrical distribution of effect sizes around the pooled estimate. The limit estimate (effect size as standard error approaches zero) was b = 1.0045 (95% CI 0.4807, 1.5282), suggesting indicating that the observed association persists even after accounting for potential bias.

### Risk of Bias

The wide-ranging JBI scores ranged from 5 to 9. No research was deemed to be of low quality. Comprehensive evidence for each included study is shown in Table 1, and supplementary file contains additional details.

## Discussion

### Relationship between digital game addiction with aggression and anger

This meta-regression revealed a statistically significant positive correlation between digital gaming addiction with aggression (r = 0.531, *p* < 0.001) and moderate correlation with anger (r = 0.348, *p* < 0.001), supporting results from earlier research studies which highlights, people who already have aggressive tendencies may be more likely to play violent or competitive video games, which reinforces hostile thought patterns.^20^ Similarly, emotional dysregulation, identified as a prominent feature of gaming addiction^2^^,^^3^ can exacerbate anger because people frequently turn to gaming as a way to avoid stressors in the real world.^19^ This reciprocal relationship, as suggested by Ferguson,^21^ is evident: aggressive people are more likely to like games that mirror their personality, and prolonged gaming may normalize aggressive approaches to problem-solving.^15^ This relationship is further supported by social learning paradigms, which portray in-game violence as a means of promoting hostility.^13^ Also, a study found that environments for games in culturally constrictive settings increase impulsivity and aggression, creating a vicious cycle of maladaptive behavior.^28^ When comparing this finding with pre-COVID-19 data, a longitudinal study^33^ showed very weak long-term correlations between aggressive video game dependency and youth aggression (r = 0.059). whereas, this meta-analysis found a stronger correlation between digital gaming addiction and anger (r = 0.348) and aggression (r = 0.531) among individuals with regional differences. Even though methodological rigor is still necessary for the accurate interpretation of these relationships, these results demonstrate the changing dynamics in the psychological and behavioral effects of gaming, where stressors related to the pandemic (dependency increased during lockdown), larger frameworks of addiction may worsen aggression and anger behaviors.

### Moderating effects

Country-level analyses regarding digital game addiction with aggression have revealed notable regional differences, with Saudi Arabia showing a significant strong correlation between aggression and gaming addiction compared to Nepal, Singapore, Italy, and Turkey which showed relatively weaker correlation. Cultural norms that are common in collectivist societies, such as the lack of leisure options and strict parental supervision, may make gaming-related annoyance and hostility worse.^28^ Teenagers in Saudi Arabia who live in restrictive environments might not have enough outlets for their emotions, which could lead to heightened violence when gaming turns becomes an escape.^28^ Western countries like Italy, on the other hand, could benefit from established regulatory frameworks that limit exposure to violent stimuli, such as age-restricted gaming material.^25^ Country-level moderation analyses with anger outcomes were marginally significant as Turkey demonstrated a stronger positive association highlighting regional disparities.

Age was not a significant mediator of the relationship between digital game addiction and aggression with no linear trend, However, it showed a slightly negative trend for anger with stronger relationships seen in younger adolescents, may be more susceptible to the emotional effects of gaming due to developmental factors such immature self-regulation.^32^ A study found that children who were addicted to gaming exhibited increased aggression and anger, which was probably caused by a lack of effective coping strategies.^30^ On the other hand, older groups showed mixed patterns, which might be because they were more independent or had a wider range of stress-reduction strategies.^25^ However, high heterogeneity highlights the impact of unmeasured factors such game genre or socioeconomic level.

The reciprocal relationship outlined in this meta-analysis raises theoretical questions: current aggressiveness may influence game selection^21^^,^^34^ and compulsive gaming may exacerbate violent behaviors by desensitizing players or excluding them from social situations.^15^^,^^35^ However, drawing conclusions about causation is impossible due to the cross-sectional character of the majority of the included studies. To Determine whether interventions reducing gaming addiction reduce aggressiveness and anger over time and elucidating temporal mechanisms require longitudinal studies.^11^^,^^12^ These findings emphasize the importance of including digital addiction awareness into nursing education curricula and ongoing professional development programs. Equipping nurses with the ability to recognize, manage, and advise persons suffering from digital gaming addiction, aggression, and anger is vital for improving patient safety and mental health outcomes. Nursing-led community awareness campaigns and school-based programs have the potential to significantly reduce the negative consequences of digital addiction on adolescent and families.^36^^,^^37^

Limitations and recommendation. This review has limitations even though it complies with PRISMA and MOOSE guidelines. First, causal interpretation is not possible with cross-sectional study designs, in order to disentangle temporal correlations, longitudinal analyses are necessary. Second, limiting inclusion to English-language research may miss cultural nuances, and using self-report tools may introduce response bias. Third, comparisons are challenging due to confounding variables (like socioeconomic status) and differences in measurement tools (like BPAQ versus SA). To validate causal mechanisms, future research must concentrate on clinical samples, standardized metrics, and experimental investigations. It is also worthwhile to look into gender differences and the style of game (violent vs. non-violent).

Conclusion. This meta-analysis demonstrates a significant correlation between digital game addiction and both aggression and anger in the post-COVID-19 era, with aggression having a strong correlate and anger having a moderate one. The study also found geographical and age-related differences, with higher associations in specific cultural situations and among younger people. These findings provide evidence of the behavioral and emotional consequences of digital gaming addiction, emphasizing the need of identifying these tendencies in clinical and public health settings. Future research and targeted treatments should expand on these findings to address this rising concern.
